# Evaluation of risk factors and treatment options associated with extra-urogenital *Mycoplasma hominis* infections

**DOI:** 10.3389/fmed.2026.1826543

**Published:** 2026-05-08

**Authors:** Xingwei Cao, Yuanli Tang, Yanping Xiao, Shumin Gu, Longhua Hu, Xinyu Liu, Yaping Hang, Yunwei Zheng, Xiuzhen Li, Qiaoshi Zhong

**Affiliations:** 1 Jiangxi Province Key Laboratory of Immunology and Inflammation, Jiangxi Provincial Clinical Research Center for Laboratory Medicine, Department of Clinical Laboratory, The Second Affiliated Hospital, Jiangxi Medical College, Nanchang University, Nanchang, Jiangxi, China; 2 Queen Mary School, Nanchang University, Nanchang, Jiangxi, China; 3 Clinical Medical College, Jiangxi University of Chinese Medicine, Nanchang, Jiangxi, China

**Keywords:** extra-urogenital infection, *Mycoplasma hominis*, quinolones, risk factors, treatment options

## Abstract

**Introduction:**

This study aimed to analyze clinical risk factors and treatment outcomes of different antibiotic regimens for extra-urogenital *Mycoplasma hominis* (*M. hominis*) infections.

**Methods:**

A retrospective cohort analysis was conducted among patients diagnosed with extra-urogenital *M. hominis* infections at a tertiary-care hospital in China from January 2018 to December 2024. Cox proportional hazards models and log-rank analysis were employed to identify independent risk factors and evaluate the clinical efficacy of antimicrobial therapeutic regimens.

**Results:**

The study included 32 patients with extra-urogenital *M. hominis* infections. Risk factors significantly associated with poor prognosis included intensive care unit (ICU) admission (*p* = 0.027), central venous catheter placement (*p* = 0.049), Sequential Organ Failure Assessment (SOFA) score (*p* < 0.001), and tracheal intubation (*p* = 0.001). Multivariate analysis identified tracheal intubation as an independent predictor of mortality. Treatment with quinolones (levofloxacin, moxifloxacin) was associated with significant reduction in white blood cell count (WBC) and C-reactive protein (CRP) levels, indicating effective control of inflammation.

**Conclusion:**

This study demonstrated that risk factors was significantly associated with prior ICU admission, tracheal intubation, SOFA score, and central venous catheter placement, with tracheal intubation identified as an independent risk factors. Quinolone antibiotics demonstrated effectiveness in reducing inflammatory markers as measured by WBC and CRP levels. These findings may provide information for the formulation of guidelines for risk assessment and treatment selection for patients with extra-urogenital *M. hominis* infections.

## 1 Introduction

*Mycoplasma hominis* (*M. hominis*), a member of the *Mycoplasmataceae* family (class Mollicutes), is a fastidious, cell wall-deficient bacterium measuring 0.3–0.4 μm in diameter (1). The absence of peptidoglycan, a characteristic feature of Mollicutes, renders it undetectable by Gram staining and intrinsically resistant to *β*-lactams and other cell wall-targeting antibiotics (2, 3). Although *M. hominis* commonly colonizes the urogenital tract in healthy individuals (21–54% of women and 4–13% of men), it can become pathogenic under certain conditions (4), These invasive manifestations typically occur in immunocompromised patients or following disruption of mucosal barriers through trauma, surgery, or invasive medical procedures (5). The diagnosis of *M. hominis* infections presents significant challenges due to its fastidious growth requirements, with conventional culture methods showing limited sensitivity and specificity. Polymerase chain reaction (PCR) has emerged as a preferred diagnostic tool, offering rapid and reliable detection even at low pathogen concentrations (6, 7, 8). While *M. hominis* is primarily associated with urogenital infections such as pelvic inflammatory disease and postpartum fever, it increasingly causes severe extra-urogenital infections, including sepsis, surgical site infections, and central nervous system infections (9, 10).

*Mycoplasma hominis* infections often remain underdiagnosed due to several factors: its slow growth characteristics, presentation with non-specific symptoms, and limited availability of routine diagnostic assays (11). Additionally, optimal antimicrobial therapy is challenging to establish due to insufficient susceptibility data. Although tetracyclines and fluoroquinolones are commonly prescribed empirically, emerging antimicrobial resistance threatens their effectiveness (9, 12). Recent reports of post-cardiopulmonary transplantation infections and multidrug-resistant strains highlight the growing clinical significance of this pathogen (10, 13).

However, several critical knowledge gaps remain in understanding *M. hominis* infections. First, the risk factors predisposing patients to invasive extra-urogenital infections are not well characterized, hampering early intervention efforts. Second, evidence-based treatment protocols, particularly for immunocompromised patients, are lacking. This study aims to address these gaps by identifying prognostic risk factors for severe *M. hominis* infections and evaluating treatment outcomes to provide information for the development of targeted management strategies.

## 2 Materials and methods

### 2.1 Study design and patient population

**Figure 1:**
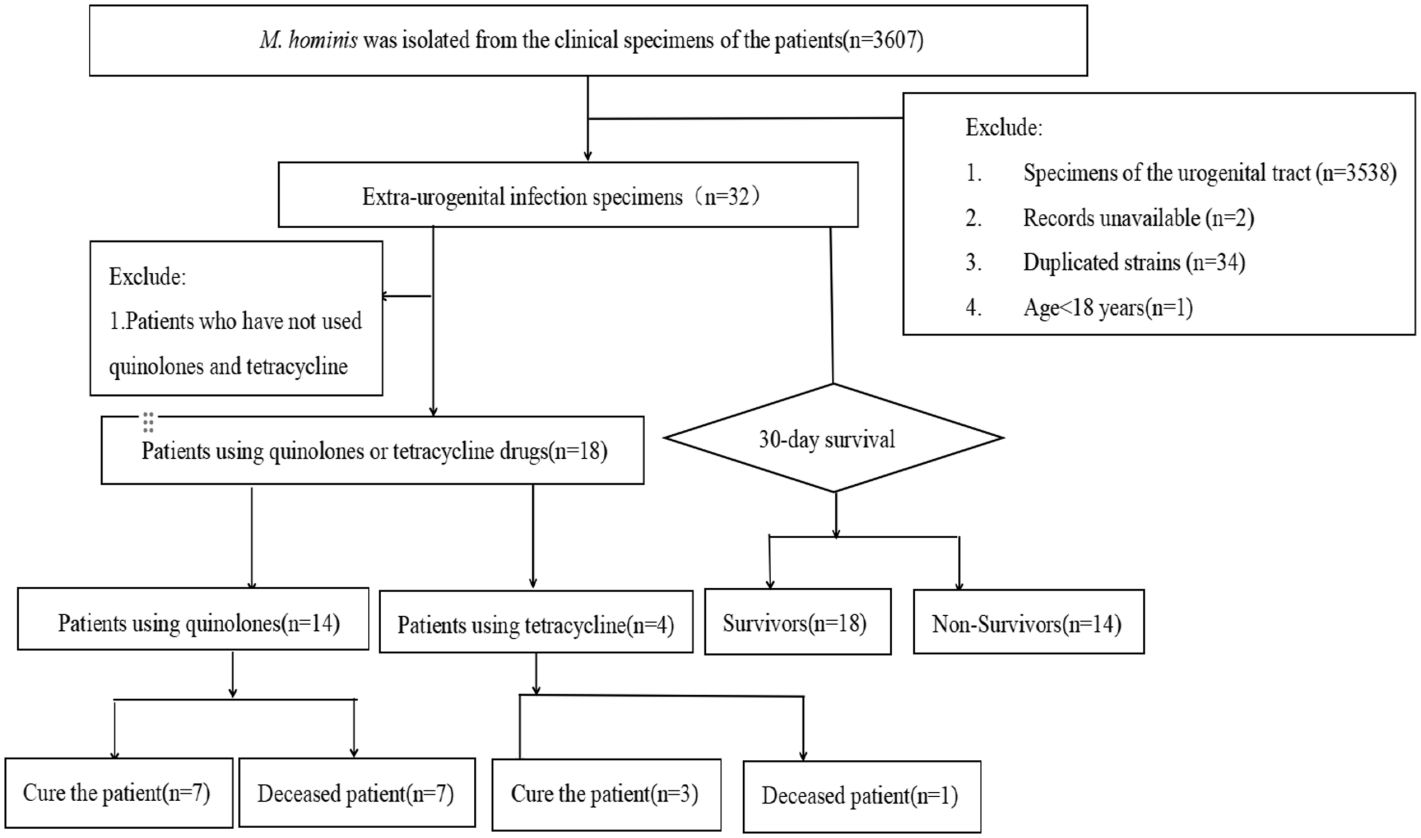
Selection process of the study population.

This retrospective study collected data on patients with extra-urogenital infection caused by *M. hominis* from January 2018 to December 2024 in a tertiary teaching hospital in southern China with approximately 4,000 beds, these patients mainly came from departments such as the infectious disease department, intensive care unit, and orthopedics. The definition of “extra-urogenital infection” specifically refers to the detection of *M. hominis* from any extra-urogenital tract sites (such as blood, respiratory tract, cerebrospinal fluid, abscesses). The patient inclusion criteria were: (1) age ≥18 years old; (2) complete clinical records and data; (3) samples were obtained from patients with extra-urogenital infection (Figure 1).

### 2.2 Data collection

The data in this study are mainly patient data obtained using our Hospital Laboratory Information System (LIS) and Hospital Information System (HIS). The data it collects mainly includes: Data such as patient gender, age, department of hospitalization, antibiotic use, endotracheal intubation, central venous catheter insertion, patient immune function status and Sequential Organ Failure Assessment (SOFA) were also collected. Blood pressure, C-reactive protein (CRP), white blood cells (WBC), granulocyte, Procalcitonin, platelet levels and other data were included at the time of infection and after 7 days of treatment for comparison. Death 30 days after infection is the primary clinical outcome.

### 2.3 Definitions

Infection is defined as the isolation of *M. hominis* from extra-urogenital specimens in patients, accompanied by at least two of the following signs and symptoms: (1) WBC or CRP levels increased; (2) body temperature greater than 38 °C; (3) heart rate >90 beats per minute; (4) respiratory rate >20 breaths per minute. In addition, if *M. hominis* is identification from non-blood specimens, it should be determined as an infection based on whether there are symptoms such as redness, swelling, heat, pain and inflammatory exudates at the identification site of the patient.

### 2.4 Microbiological analysis

The *M. hominis* was identification using the Smart MS 5020 (DL, China) or MALDI-TOF MS (biomacriux, France) and molecular methods (PCR). Antimicrobial susceptibility testing was conducted using a commercially available broth-based phenotypic susceptibility kit (Lizhu, China) in accordance with the manufacturer’s instructions.

### 2.5 Statistical analysis

The Shapiro–Wilk test was applied to assess the normality of continuous variables. Normally distributed data were presented as mean ± standard deviation (SD), while categorical variables were summarized as cumulative frequencies and percentages. Non-normally distributed continuous variables were analyzed using the Mann–Whitney U test, whereas categorical variables were compared via the chi-square test. Cox regression analysis was employed to identify independent risk factors influencing prognosis, and log-rank analysis was utilized to evaluate the association between different antibacterial regimens and 30-day mortality. Changes in inflammatory markers before and after antibacterial therapy were examined using paired *t*-tests. All statistical analyses were performed with SPSS software (version 25.0), and a two-tailed *p*-value < 0.05 was considered statistically significant.

## 3 Results

### 3.1 Demographic characteristics of patients with extra-urogenital *Mycoplasma hominis* infections

Among the 32 patients included in this study, males constituted 78.1% (25/32) of the cohort, Half of the patients had undergone surgery (50.0%, 16/32), and 37.5% (12/32) had prior ICU admission. The most common invasive procedures during hospitalization were drainage tube (59.4%, 19/32) and tracheal intubation (62.5%, 20/32). Cerebrovascular disease was the most prevalent underlying condition (28.1%, 9/32), while hypertension was the most common comorbidity (53.1%, 17/32). Detailed patient characteristics are presented in Table 1.

**Table 1:** Characteristics of patients with extra-urogenital infection of *M. hominis.*

Characteristics	Total *N* = 32	Survival *N* = 18	Mortality *N* = 14	*p* value
Age (years), mean ± SD	55.7 ± 15.1	54.2 ± 15.6	57.6 ± 14.7	0.535
Male	25 (78.1%)	14 (77.8%)	11 (78.6%)	1.000
*M. hominis* identified on blood samples	25 (78.1%)	12 (66.7%)	13 (92.9%)	0.104
ICU stay prior to infect	12 (37.5%)	3 (16.7%)	9 (64.3%)	0.027
Hospital stay >30 days prior to infect	5 (15.6%)	3 (16.7%)	2 (14.3%)	1.000
Surgical operation	16 (50.0%)	11 (61.1%)	5 (35.7%)	0.156
Hemodialysis	2 (6.3%)	1 (5.6%)	1 (7.1%)	1.000
Illness severity at time of BSI				
SOFA (IQR)	4.5 (1–7)	2 (0–4.5)	7 (5.85–9.3)	<0.001
Invasive procedure				
Urinary catheterization	5 (15.6%)	3 (16.7%)	2 (14.3%)	1.000
Gastrointestinal catheterization	4 (12.5%)	1 (5.6%)	3 (21.4%)	0.319
Central venous catheterization	9 (28.1%)	2 (11.1%)	7 (50.0%)	0.049
Drainage tube	19 (59.4%)	13 (72.2%)	6 (42.9%)	0.280
Tracheal intubation	20 (62.5%)	7 (38.9%)	13 (92.9%)	0.001
Antimicrobial exposure within 30 days				
Piperacillin-tazobactam	15 (46.9%)	6 (33.3%)	9 (64.3%)	0.074
Cephalosporin	5 (15.6%)	3 (16.7%)	2 (14.3%)	1.000
Fluoroquinolone	2 (6.3%)	1 (5.6%)	1 (7.1%)	1.000
Cefoperazone-sulbactam	2 (6.3%)	2 (11.1%)	0 (0.0%)	0.486
Underlying disease				
Cerebrovascular diseases	9 (28.1%)	4 (22.2%)	5 (35.7%)	0.699
Liver disease	2 (6.3%)	2 (11.1%)	0 (0.0%)	0.486
Malignant tumor	1 (3.1%)	0 (0.0%)	1 (7.1%)	0.469
Diabetes	10 (31.3%)	5 (27.8%)	5 (35.7%)	0.450
Immunocompromised status	2 (6.3%)	1 (5.6%)	1 (7.1%)	1.000
Comorbid conditions				
Hypoproteinemia	17 (53.1%)	10 (55.6%)	7 (50.0%)	1.000
Septic shock	2 (6.3%)	1 (5.6%)	1 (7.1%)	0.212
Acute cerebral hemorrhage	3 (9.4%)	2 (11.1%)	1 (7.1%)	1.000
MOF	2 (6.3%)	1 (5.6%)	1 (7.1%)	1.000
Gastrointestinal bleeding	1 (3.1%)	0 (0.0%)	1 (7.1%)	0.452
Acute pancreatitis	1 (3.1%)	0 (0.0%)	1 (7.1%)	0.452

ICU, intensive care unit; BSI, bloodstream infection; SOFA, sequential organ failure assessment; IQR, interquartile range; MOF, multiple organ failure.

### 3.2 Risk factors for 30-day mortality in extra-urogenital *Mycoplasma hominis* infections

The overall 30-day mortality rate was 43.8% (14/32). Cox regression analysis identified tracheal intubation as an independent risk factors for mortality [*p* = 0.039, OR (95% CI): 0.113 (0.014–0.592)]. Detailed results are shown in Table 2.

**Table 2:** Analysis of the risk factors for 30-day mortality in patients with extra-urogenital infection of *M. hominis.*

Variable	Survival (*n* = 18)	Mortality (*n* = 14)	Multivariable analysis	
			*p* value	OR (95% CI)
Age (≥ 60)	7 (38.9%)	8 (57.1%)	0.456	1.497 (0.519–4.321)
*M. hominis* identified on blood samples	12 (66.7%)	13 (92.9%)	0.339	2.735 (0.348–21.516)
Tracheal intubation	7 (38.9%)	13 (92.9%)	0.039	0.113 (0.014–0.892)

### 3.3 Impact of different antimicrobial regimens on 30-day mortality

**Figure 2:**
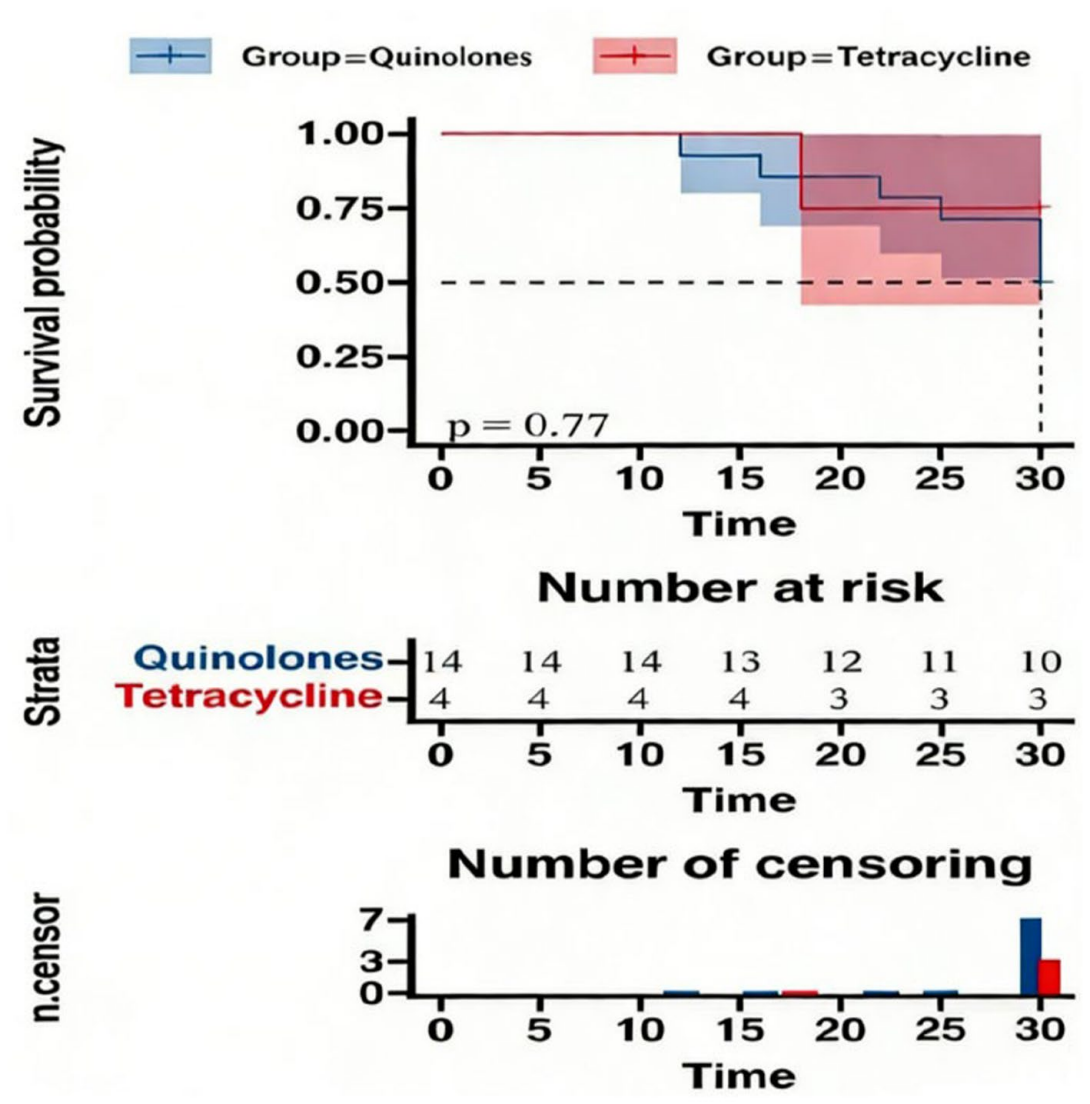
Effect of different effective antimicrobial regimens on 30-day mortality in infected patients.

Treatment options for *M. hominis* infections primarily include macrolides, quinolones, and tetracyclines, as recommended by expert consensus. In our cohort, only 18 of the 32 patients received either quinolones or tetracyclines. Among 14 patients treated with quinolones, 7 patients achieved clinical cure, while 3 of 4 patients treated with tetracyclines were cured. However, the difference in cure rates between these antibiotic classes was not statistically significant (*p* = 0.77) (Figure 2).

### 3.4 Effects of antimicrobial treatment on inflammatory markers

**Figure 3:**
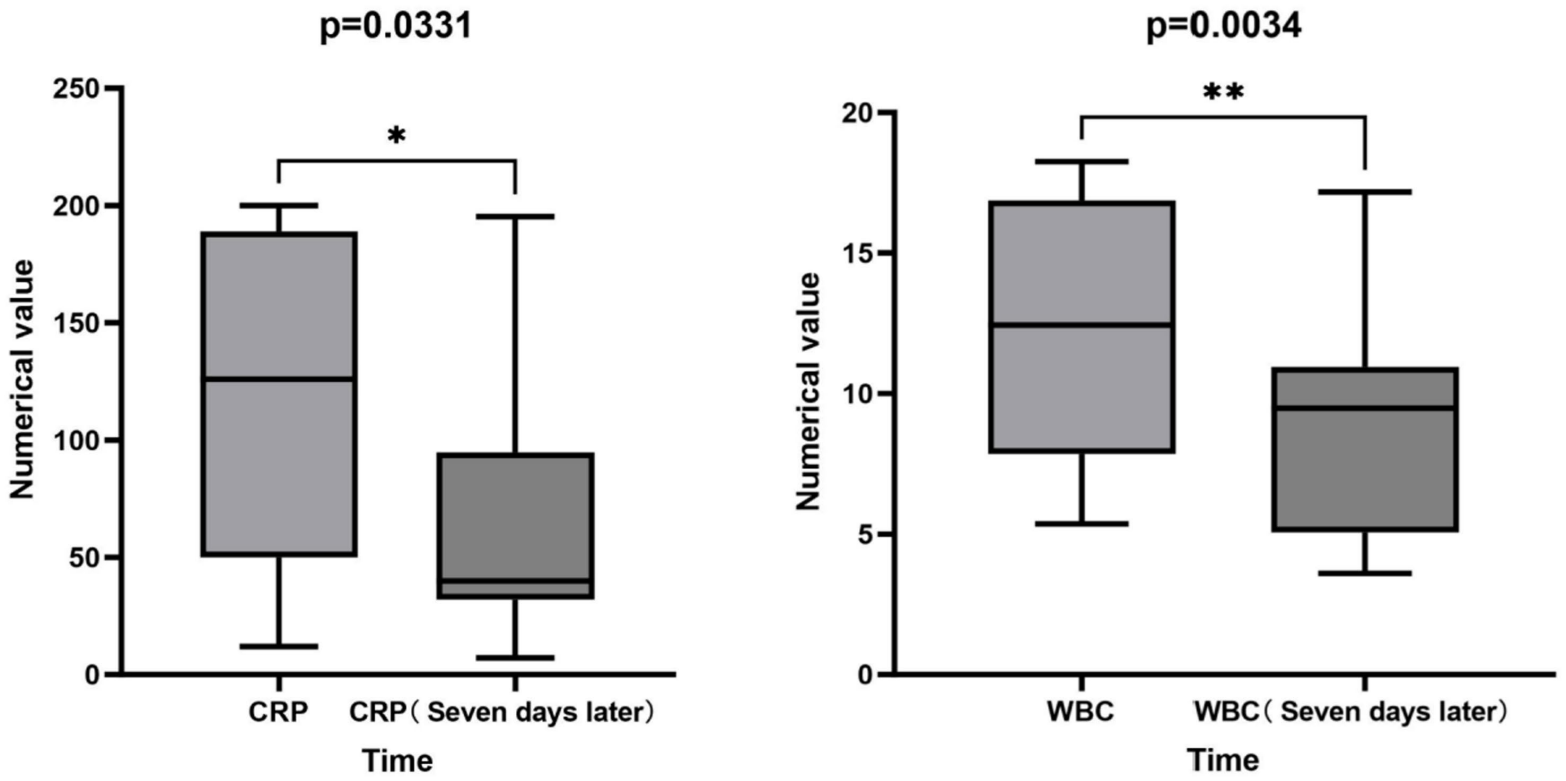
Analysis chart of inflammatory indicators before and after the use of quinolone antibiotics.

Analysis of inflammatory markers before and after antibiotic treatment focused on changes in Procalcitonin, WBC and CRP levels. Due to the limited number of patients receiving tetracyclines and the data of 3 patients using quinolones are incomplete. Analysis was conducted on 11 patients treated with quinolones. Paired *t*-tests comparing inflammatory markers at baseline and 7 days post-treatment revealed significant reductions in WBC (*p* = 0.0034) and CRP levels (*p* = 0.0331). No significant changes were observed in Procalcitonin levels (Figure 3).

## 4 Discussion

*Mycoplasma hominis* is a ubiquitous urogenital colonizer, detected in 4–13% of men and 21–54% of women in asymptomatic populations (4). Although traditionally associated with conditions such as pelvic inflammatory disease and urethritis, its role in extra-urogenital infections—including pneumonia, septic arthritis, and subdural empyema—is increasingly recognized (13). It is worth noting that the colonization rate of *M. hominis* in the respiratory tract of patients with respiratory-related diseases is 8%, while in healthy individuals it is 1–3%. This indicates that the colonization rate is also related to an individual’s immune status (12). Of the 18 species in humans, only *Ureaplasma urealyticum*, *Ureaplasma parvum*, *Mycoplasma hominis* and *Mycoplasma genitalium* are established pathogens, which are linked to urogenital tract infections (13). Due to the lack of a cell wall, *M. hominis* is difficult to detect by gram staining and it is not easy to obtain colonies through culture (14, 15), which leads to the inability of experiments to confirm the infection of the pathogen and often delays clinical treatment (16). This study analyzes the related risk factors of the extra-urogenital *M. hominis* Infections. Furthermore, we also evaluated the differences in therapeutic effects among different antibacterial drugs and the efficacy of antidrugs on patients before and after use. Our findings emphasize the need for further large-scale prospective studies to provide information for the development of guidelines for the prevention, diagnosis and treatment of extra-urogenital *M. hominis* Infections.

Our study revealed distinct demographic and clinical patterns in extra-urogenital *M. hominis* infections. Male patients constituted the majority of cases, and nearly half of the patients were over 60 years old, consistent with previously reported demographics (17). Although *M. hominis* colonization rates in the urogenital tract are typically higher in females, our study found a higher rate of extra-urogenital infections in males, this is also consistent with the results previously reported (18, 19). This gender disparity may be attributed to several factors: (1) the anatomical structure of the male urethra, which while less conducive to colonization, may facilitate ascending infection; (2) potentially more intense immune responses to invasion in males, leading to more pronounced clinical symptoms; (3) and diagnostic criteria based on symptomatic presentation, which may preferentially capture male cases due to their higher likelihood of seeking medical attention for symptoms.

Other key clinical characteristics included cerebrovascular disease as the most common underlying condition (28.1%, 9/32), with hypertension being the predominant complication (53.1%, 17/32). Half of the patients had undergone surgery, and 37.5% had experienced ICU admission prior to infection. The most frequent invasive procedures during hospitalization were catheterization (59.3%, 19/32) and tracheal intubation (62.5%, 20/32). The high prevalence of severe underlying conditions, recent surgery, and immunocompromised states among infected patients suggests these factors may increase susceptibility to *M. hominis* infection. This observation aligns with previous studies documenting extra-urogenital *M. hominis* infections in post-surgical cases, immunocompromised patients, and those with abscesses, central nervous system infections, and bone/joint infections (16, 20, 21).

Our analysis identified significant associations between mortality and several clinical factors: elevated SOFA scores, prior ICU stays, central venous catheterization, and tracheal intubation. These correlations likely reflect the combined impact of immunosuppression from severe underlying conditions, prolonged hospitalization, and increased pathogen exposure through invasive procedures. This mechanistic relationship is supported by current understanding of *M. hominis* clearance dynamics – while phagocytes and macrophages in the reticuloendothelial system typically eliminate circulating pathogens, this process is often impaired in critically ill patients (22).

Our multivariate analysis identified tracheal intubation as an independent risk factor for 30-day mortality in patients with extra-urogenital *M. hominis* infections. This association remained significant after adjusting for potential confounders, suggesting that the intubation procedure or its sequelae directly contribute to poor outcomes, beyond merely serving as a marker of patient frailty. Notably, tracheal intubation has also been identified as a risk factor for developing extra-urogenital *M. hominis* infections. While physical trauma to the respiratory tract during intubation likely compromises the immune barrier and facilitates infection by colonizing *M. hominis*, several additional mechanisms may contribute to adverse outcomes (23). These include biofilm formation on the endotracheal tube, disruption of the local microbiome and mucociliary clearance, and the immunomodulatory effects of the foreign body and associated inflammation (24). Critically, an established *M. hominis* respiratory tract infection in an intubated patient creates a significant risk for hematogenous dissemination or local spread to other extra-urogenital sites (25, 26), a risk compounded by the frequent presence of other invasive devices, critical illness, and potential immunosuppression in this population. Therefore, we emphasize the importance that clinicians should strictly adhere to aseptic techniques, meticulous oral care, and minimize the intubation time as much as possible during intubation and subsequent airway management. Further research is also needed to confirm these associations and clarify the exact pathogenic mechanisms that link intubation to the adverse consequences of *M. hominis* infection. Concurrently, variations in post-intubation anti-infection treatment strategies and their effects on clinical outcomes deserve thorough exploration. Adequate therapeutic drug concentrations in lung tissue and airway secretions may critically determine the prognosis of intubated patients. Hence, future studies should extend beyond the physical impact of intubation to systematically assess pathogen resistance patterns, the timing of individualized anti-infective therapy, and the comparative advantages and disadvantages of local versus systemic combination treatments.

Due to the absence of a cell wall in mycoplasma, antibiotic options are limited. Treatment is restricted to antibiotics that inhibit DNA replication (such as fluoroquinolones) and those that interfere with protein synthesis (such as macrolides and tetracyclines) (27). The prevalence rate and antibiotic sensitivity profile vary by region, influenced by local antibiotic usage and previous exposure history (28). In recent years, antibiotic resistance has increased, primarily due to inappropriate antibiotic use (29). This resistance can develop through genetic mutations or the acquisition of resistance determinants (30, 31, 32). According to expert consensus, the primary recommended treatments for human mycoplasma infections are macrolides, fluoroquinolones, and tetracyclines (33). Among 18 patients treated with either quinolones or tetracyclines, we found no significant difference in cure rates between the two antibiotic classes, While our findings are limited by the small sample size, they suggest the need for larger-scale research comparing the antimicrobial efficacy of these agents. Given the similar therapeutic effects observed, antibiotic selection should be based on individual patient factors and contraindications. Clinical reports indicate that tetracyclines and quinolones pose teratogenic risks in pregnant women with *M. hominis* infections. Consequently, macrolide antibiotics, particularly azithromycin and erythromycin, remain the primary treatment option for mycoplasma infections during pregnancy (34).

Pathogenic infections typically trigger stress responses in the body, including changes in inflammatory markers such as WBC, neutrophil, and CRP levels, Although *M. hominis* is considered an atypical pathogen, it can similarly affect these inflammatory indicators (22, 35). In our analysis, we focused on examining inflammatory marker changes in 11 patients treated with quinolone antibiotics. It was found that only the changes in WBC and CRP levels were statistically significant, This result aligns with previous studies showing that WBC and CRP typically increase during *M. hominis* infection, other inflammatory markers show no significant changes (22). Notably, however, the change in neutrophil count often a hallmark of acute bacterial infection did not reach statistical significance in our study. This discrepancy might suggest a distinct inflammatory response pattern induced by *M. hominis*, potentially differing from classic pyogenic bacteria, perhaps involving different cellular pathways or a more chronic inflammatory profile. These results suggest that elevated WBC and CRP levels may serve as a useful diagnostic indicator for *M. hominis* infection (21, 36, 37), However, their high non-specificity must be emphasized; elevations are common to numerous infectious and non-infectious inflammatory conditions. Therefore, they cannot be diagnostic alone and must be interpreted within the full clinical context, alongside epidemiological risk factors and confirmed by specific microbiological tests. Similarly, the dynamic changes in WBC and CRP may offer valuable adjunctive information for monitoring the response to quinolone treatment.

The present study has several limitations that warrant consideration. First, the relatively small sample size and incomplete patient data may compromise the statistical power and generalizability of the findings, especially when conducting multivariate analysis and subgroup comparisons. As a retrospective study, due to the insufficient sample size and missing information, it has inherent selection bias and information bias, these methodological limitations reduce the reliability of the conclusion. Second, the single-center design restricts the diversity of the cohort and may not account for regional or institutional variations in patient demographics, clinical practices, or pathogen prevalence, thereby reducing the external validity of the results. Third, the absence of *M. hominis* antimicrobial susceptibility testing (AST) data hinders a comprehensive evaluation of treatment efficacy, particularly in guiding evidence-based antibiotic stewardship for infections involving this pathogen. Therefore, our findings are preliminary and of an exploratory nature, and require further large-scale prospective studies to provide information for the formulation of guidelines for the prevention, diagnosis and treatment of extragenital mycoplasma infections in the urogenital tract in the future.

## 5 Conclusion

Our study provide information on several factors significantly associated with mortality in patients with extra-urogenital *M. hominis* infections: prior ICU admission, tracheal intubation, central venous catheterization, and elevated SOFA scores. Among these, multivariate analysis identified tracheal intubation as an independent risk of mortality. Additionally, our findings demonstrated that treatment with quinolone antibiotics effectively reduced inflammatory markers, as evidenced by significant decreases in WBC counts and CRP levels.

## Data Availability

The original contributions presented in the study are included in the article/supplementary material, further inquiries can be directed to the corresponding author.
